# Supplementary material for: A non-canonical BRD9-containing BAF chromatin remodeling complex regulates naive pluripotency in mouse embryonic stem cells
Source: Nat Commun. 2018 Dec 3;9:5139. doi: 10.1038/s41467-018-07528-9 (PMC6277444; doi:10.1038/s41467-018-07528-9)
Supplement: Supplementary file 1 — Supplementary Information [file 41467_2018_7528_MOESM1_ESM.pdf]

Supplementary Information for Gatchalian, et al.:

A non-canonical BRD9-containing BAF chromatin remodeling complex regulates naïve pluripotency in mouse embryonic stem cells

Supplementary Figures

Supplementary Figure 1: Phenotypic effects of BRD9 inhibition/knockdown or BRG1 inhibition

Supplementary Figure 2: GBAF complex characterization in HCT116 cells

Supplementary Figure 3: Effect of I-BRD9 on GBAF complex assembly and chromatin binding

Supplementary Figure 4: Characterization of BRD4's role in GBAF's function

Supplementary Tables

Supplementary Table 1: List of BRD9-interacting proteins in mouse ESCs

Supplementary Table 2: List of primer sequences used for RT qPCR

Supplementary Figure 1

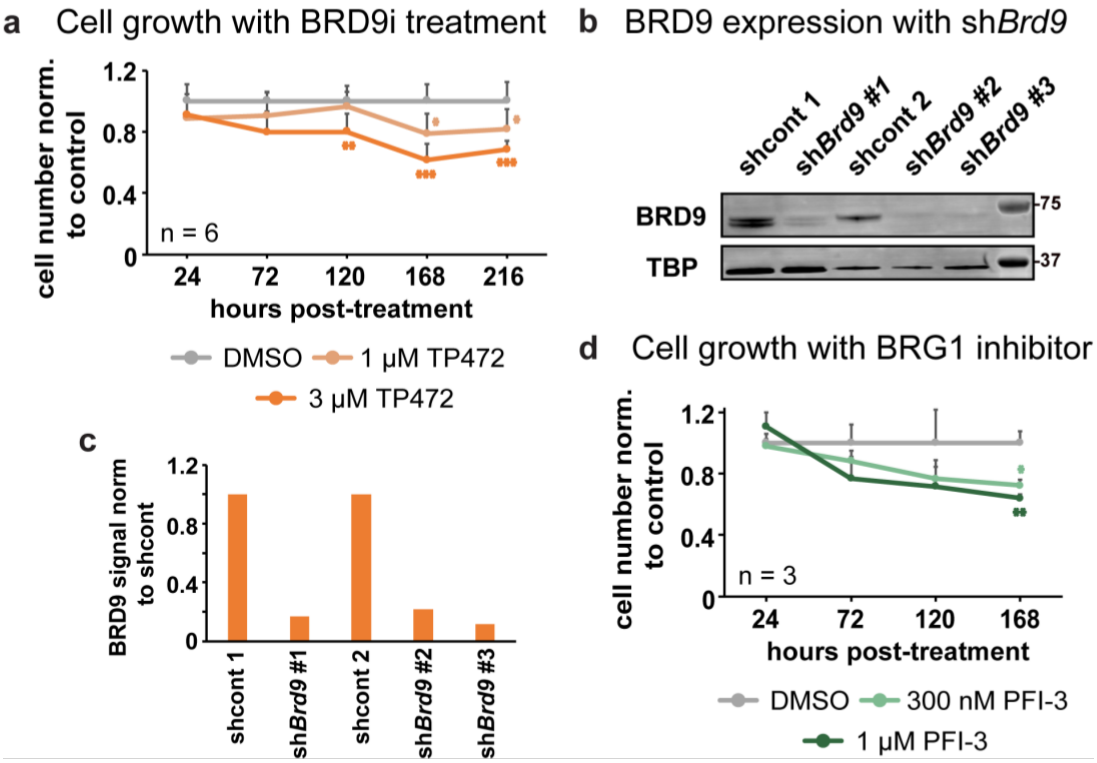

Supplementary Figure 1. a) Time course experiment assessing mouse ES cell number after treatment with DMSO or TP472 at either 1 or 3 µM. Error bars represent one standard deviation from the mean of biological replicates. Two-tailed t-test was performed to obtain the p-values, n = 6. * $p < 0.05$, ** $p < 0.01$, *** $p < 0.001$. **b)** Immunoblotting analysis of BRD9 protein expression in nuclear lysates after mouse ESCs were transduced with shRNA against a scrambled control or three independent shRNAs against *Brd9*. Shcontrol 1 is used for sh*Brd9* #1 (both in pGipZ) and shcontrol 2 is used for sh*Brd9* #2 and #3 (all in SMARTvectors). TATA-binding protein (TBP) was used as a loading control. Molecular weights from ladder are indicated. **c)** Quantification of immunoblotting in b, first normalized to TBP then to appropriate shcontrol. **d)** As in a, except ESCs were treated with either DMSO or PFI-3 at 300 nM or 1 µM; n = 3. Source data for a-d are provided as Source Data file.

Supplementary Figure 2

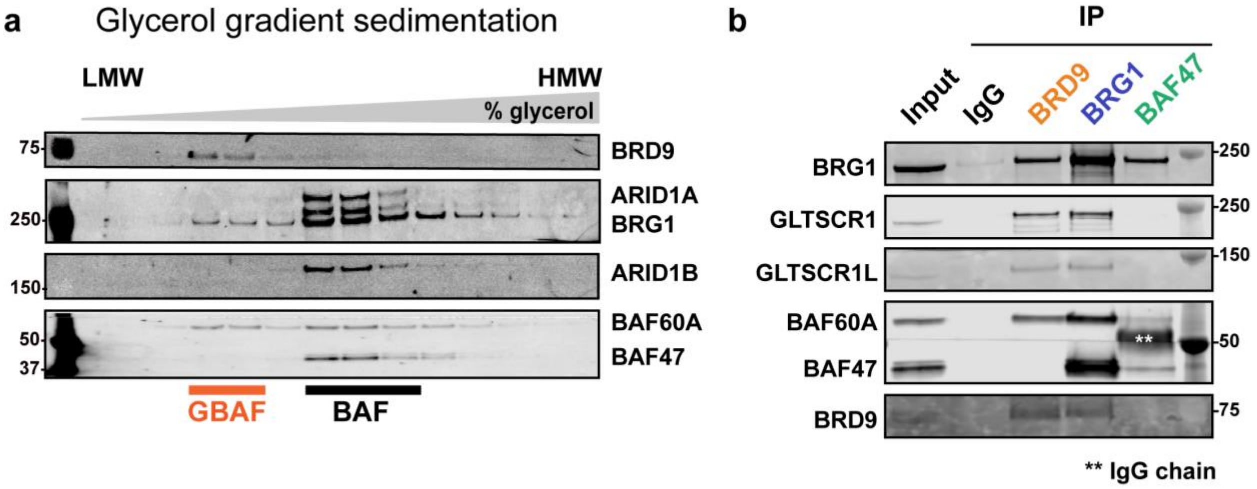

Supplementary Figure 2. a) Immunoblotting analysis of fractions after HCT116 nuclear lysates were subjected to a density sedimentation assay in 10-30% glycerol gradient. LMW and HMW indicate lower and higher molecular weights, respectively. Molecular weights from ladder are indicated in red. **b)** IP experiments from HCT116 nuclear lysates using antibodies against IgG, BRG1, BRD9 and BAF47. IgG chain is marked with double asterisks. Source data are provided as Source Data file.

Supplementary Figure 3

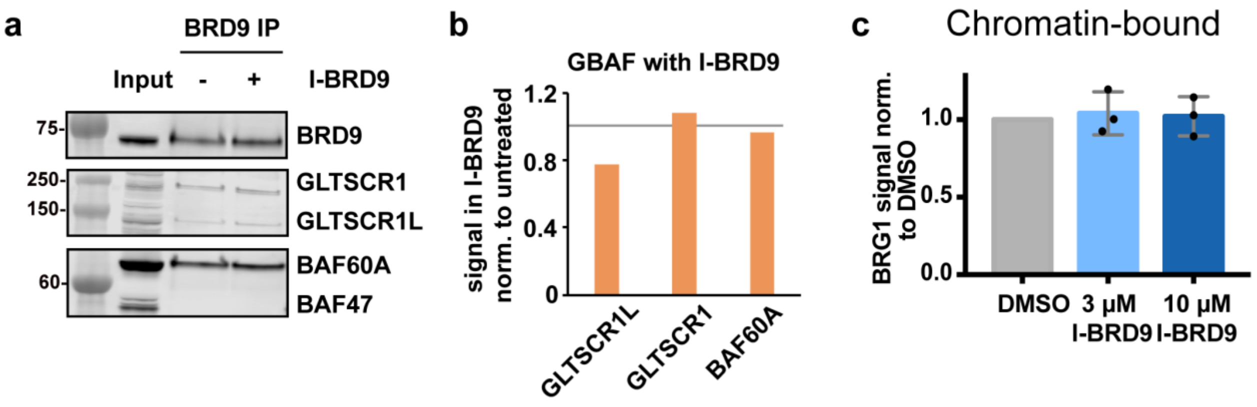

Supplementary Figure 3. a) IP experiments from mouse ESC nuclear lysates using BRD9 antibody with or without I-BRD9. Molecular weights from ladder are indicated. **b)** Quantification of western blot signal for GBAF subunits first normalized to BRD9 bait signal then normalized to the signal in the untreated sample. Expression in untreated sample is indicated with a gray line. **c)** Quantification of BRG1 chromatin fraction signal from Figure 6a normalized to the loading control, Histone H3, then normalized to DMSO sample. Average of three independent experiments; error bars represent one standard deviation from the mean. Source data are provided as Source Data file.

Supplementary Figure 4

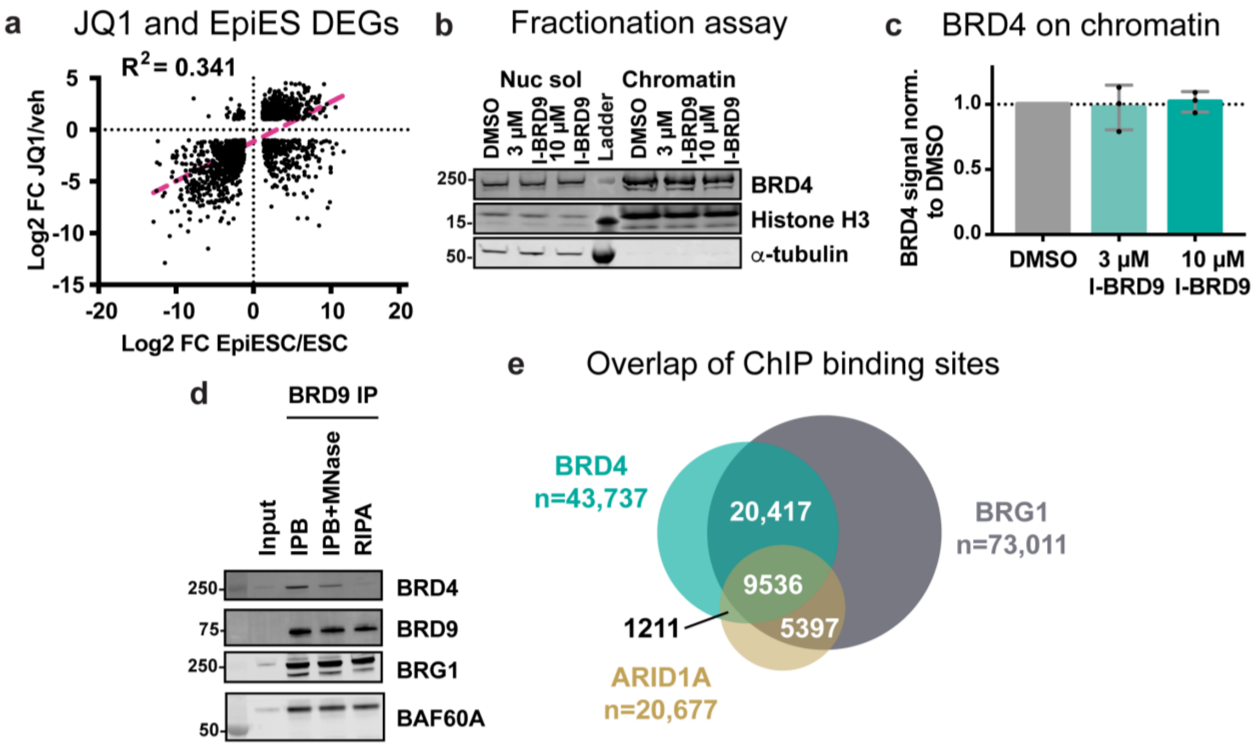

Supplementary Figure 4. a) Scatterplot of the mRNA log2 FCs in JQ1/vehicle and EpiESC/ESC for 1666 common DEGs. Linear regression analysis was performed to calculate the R^2, with the best fit shown as a pink dashed line. **b)** Representative immunoblotting analysis of a cellular fractionation assay in mouse ESC lysates after treatment with either DMSO or I-BRD9 at 3 or 10 µM for 24 hours. Molecular weights from ladder are indicated. **c)** Quantification of BRD4 chromatin fraction signal normalized to the loading control, Histone H3, then normalized to DMSO sample. Average of three independent experiments; error bars represent one standard deviation from the mean. **d)** IP experiments from mouse ESC nuclear lysates using BRD9 antibody and washed with buffers of varying stringency (increasing from left to right.) IP = wash buffer; MNase = Micrococcal Nuclease; RIPA = higher stringency wash buffer.

See the Source Data File for original scans that include molecular weights. **e)** Venn diagram of overlap between BRG1, BRD4 and ARID1A ChIP sites, with n representing the number of observed peaks. Source data for b, c, and d are provided as Source Data file.

Supplementary Table 1. List of statistically significant proteins in the BRD9 IP-Mass Spectrometry experiment.

Protein ID	AC test p value	Fold Change (BRD9/IgG)
BAF155/SMARCC1	1.00E-05	208
GLTSCR1L/BICRAL	1.00E-05	185
BAF60A/SMARCD1	1.00E-05	170
BRM/SMARCA2	1.00E-05	169
BRG1/SMARCA4	1.00E-05	133
VIM	1.00E-05	114
BRD9	1.00E-05	110
BAF53A/ACTL6A	1.00E-05	78
GLTSCR1/BICRA	1.00E-05	75
BAZ1B	1.00E-05	46
NVL	1.00E-05	39
PRSS1	1.00E-05	8.11
SMARCA5	1.11E-05	27
RBPMS2	3.43E-05	34
GM17087	0.000359589	26
KRT18	0.000647042	24
HSP90AA1	0.000867951	23
KRT31	0.003769715	18
KRT32	0.003769715	18
KRT35	0.003769715	18
KRT33B	0.003769715	18
KRT40	0.003769715	18
KRT6A	0.004691553	5.47
KRT28	0.005056747	17
KRT24	0.005056747	17
KRT36	0.005056747	17
ERH	0.006169763	23
GM5414	0.006783189	16
BCL7B	0.006783189	16
HSP90AB1	0.007979874	22
MDN1	0.009099063	15
KRT6B	0.010400801	5.15
BCL7A	0.012205606	14
NPM1	0.012205606	14
BCLAF1	0.016372767	13
RBM14	0.016372767	13
HIST1H4A	0.018380452	7.6
BCL7C	0.021962652	12
SMARCA1	0.028223324	17
SAP18	0.029461	11
SS18	0.029461	11
NOP56	0.029461	11
ACIN1	0.035903108	10.5
KRT90	0.038966593	6
KRT72	0.039519387	11
KRT78	0.039519387	11
RPS4X	0.039519387	11
KRT10	0.046485689	2.08
HNRNPD	0.04904715	4.47

Supplementary Table 2. List of primer sequences used for RT qPCR.

Gene	Forward	Reverse
Esrrb	TTTCTGGAACCCATGGAGAG	AGCCAGCACCTCCTTCTACA
Klf4	CGGGAAGGGAGAAGACACT	GAGTTCCTCACGCCAACG
Pou5f1	ACATCGCCAATCAGCTTGG	AGAACCATACTCGAACCACATCC
Sox2	CATGAGAGCAAGTACTGGCAAG	CCAACGATATCAACCTGCATGG
Nanog	AAGATGCGGACTGTGTTCTC	CGCTTGCACTTCATCCTTTG
Gapdh	CACTCTTCCACCTTCGATGCC	CCTTGGAGGCCATGTAGGCC
